# Antiviral Therapy for a Postpartum Flare in Women with Chronic HBV Infection Shortens the ALT Recovery Time and Reduces Hepatitis Re-Flare Rates within 4 years

**DOI:** 10.1155/2022/4753267

**Published:** 2022-06-20

**Authors:** Min Quan, Cong Liu, Wei Li, Hui-Chun Xing

**Affiliations:** ^1^Center of Liver Diseases Division 3, Beijing Ditan Hospital, Capital Medical University, Bejing 100015, China; ^2^Peking University Ditan Teaching Hospital, Beijing 100015, China; ^3^Department of Infectious Diseases, The Second Hospital of Shanxi Medical University, Taiyuan, Shanxi 030000, China

## Abstract

**Background:**

Few studies explored whether anti-hepatitis B virus (HBV) therapy should be initiated during postpartum hepatitis flare.

**Aim:**

This study aimed to analyze the effect of anti-HBV therapy on postpartum hepatitis flare and evaluate the prognosis within 4 years postpartum.

**Methods:**

This retrospective study enrolled hepatitis B surface antigen (HBsAg)-positive and hepatitis B e antigen (HBeAg)-positive pregnant women with HBV DNA ≥ 10^6^ IU/mL. A total of 152 pregnant women were included: 103 in the prophylactic anti-HBV therapy group (PT-G) and 49 in the non-prophylactic anti-HBV therapy group (NPT-G). The women with a postpartum flare were assigned to the anti-HBV therapy group (AT-G) and non-anti-HBV therapy group (NAT-G) to analyze the effect of postpartum anti-HBV therapy on hepatitis flare. Virological and biochemical parameters were assessed.

**Results:**

Taking postpartum 12 weeks as the cutoff point, the ALT recovered time for postpartum flare women is shorter in AT-G (*n* = 16, 42.1%) or PT-G (*n* = 23, 34.8%) than in NAT-G (*n* = 14, 23.0%; *x*^2^ = 4.067, *P*=0.044) or NPT-G (*n* = 4, 11.1%; *x*^2^ = 5.579, *P*=0.018). Taking postpartum 26 weeks as the cutoff point, the ALT recovered time is shorter in AT-G (*n* = 35, 57.3%) or PT-G (*n* = 44, 66.7%) than in NAT-G (*n* = 32, 84.2%; *x*^2^ = 7.707, *P*=0.006) or NPT-G (*n* = 16, 44.4%; *x*^2^ = 4.749, *P*=0.029). Postpartum flare recovery time was positively correlated with HBV DNA level at delivery [*r* = 0.223, *P*=0.025, 95%CI (0.022～0.41)]. The hepatitis re-flare rates within postpartum 4 years in AT-G (*n* = 3, 9.68%) is lower than that in NAT-G (*n* = 24, 45.4%; *x*^2^ = 14.003, *P* ≤ 0.001). The HBeAg, HBsAg, HBV DNA, and ALT level at postpartum 4 years in AT-G were lower than that in NAT-G (*P* < 0.001).

**Conclusion:**

Anti-HBV therapy for postpartum hepatitis flare of women with chronic HBV could shorten the ALT recovery time and reduce hepatitis re-flare rates within 4 years of postpartum.

## 1. Introduction

The positive rate of HBsAg in pregnant women in China is about 6–8%. Infantile infection of HBV is the main cause of chronic hepatitis B, and mother-to-child transmission (MTCT) is the main mode of infantile infection with hepatitis B virus [1]. Prophylactic anti-HBV therapy in the third trimester of pregnancy significantly reduced the incidence of mother-to-child transmission of HBV in mothers with high viremia [2, 3]. However, postpartum hepatitis flared in 25–44.7% [4–7] of HBV-infected women. The factors related to postpartum hepatitis flare are complicated. It is not easy to identify whether postpartum hepatitis flare and HBV are correlated. Some hormones in pregnant women induce immune changes during pregnancy. The current hypothesis postulates that the reconstitution of the immune system may be responsible for the high incidence of postpartum hepatitis flare. Also, the destruction of immune balance may cause the replication of HBV, which may induce hepatitis flare. Studies have shown that postpartum hepatitis flare is often mild and most spontaneously resolves [8–10]. However, severe cases have also been reported [11], Severe flares necessitating liver transplantation when salvage anti-HBV therapy fails have been rarely reported [11–13]. Some studies showed that extending anti-HBV therapy did not protect against postpartum flares or affect hepatitis B e antigen (HBeAg) seroconversion rates [8]. Liu et al. reported that it was safe for most women to withdraw the use of telbivudine after delivery, whereas activating serological response encouraged extended anti-HBV therapy for mothers with ALT elevation during pregnancy [7]. However, studies exploring whether immediate anti-HBV therapy is recommended for women with postpartum hepatitis flare, as well as the efficacy evaluation after anti-HBV treatment and prognosis evaluation, are limited.

In fact, the onset of a postpartum flare may represent an opportunity for anti-HBV treatment. This study aimed to evaluate the effect of anti-HBV therapy initiated after postpartum hepatitis flare on the recovery of liver inflammation and the occurrence of hepatitis re-flare. Meanwhile, the effect of prophylactic anti-HBV treatment on the recovery time of postpartum hepatitis flare was evaluated. Our secondary objective was to assess the levels of ALT, HBV DNA, hepatitis B surface antigen (HBsAg), and HBeAg in women for 4 years postpartum.

## 2. Methods

Pregnant women with serum HBsAg positive and a high HBV viral load (≥10^6^ IU/mL) were retrospectively recruited from Beijing Ditan Hospital between August 2016 and September 2017. They were divided into the prophylactic anti-HBV therapy group (PT-G, 103/152) (telbivudine (LdT) 600 mg/day or tenofovir (TDF) 300 mg/day) and the non-prophylactic therapy anti-HBV group (NPT-G, 49/152) based on whether they received anti-HBV therapy during the third trimester of gestation. A total of 103 women with alanine aminotransferase (ALT) flare (ALT > 40 U/L) within 26 weeks postpartum, were assigned to the anti-HBV therapy group postpartum (AT-G, 38/103) and the non-anti-HBV therapy group postpartum (NAT-G, 65/103) based on whether they accepted anti-HBV therapy within 26 weeks postpartum or not. Postpartum hepatitis flare was defined as ALT elevation >40 U/L in the first 26 weeks after delivery. ALT recovery was defined as ALT returning to ≤40 U/L. Hepatitis re-flare was defined as ALT > 40 U/L after the postpartum ALT flare was restored to ≤40 U/L.

All participants were screened for the following eligibility criterion: seropositive for both HBsAg and HBeAg, HBV DNA levels ≥ 10^6^ IU/mL, and ALT < 1 × upper limit of normal (ULN; 40 U/L). The patients with any of the following conditions were excluded: (1) coinfection with hepatitis C, D, E, or human immunodeficiency virus (HIV); (2) evidence of hepatocellular carcinoma, cirrhosis, or any other disease that could influence the study results; and (3) concurrent treatment with immune modulators, cytotoxic drugs, or steroids.

The serum levels of HBV DNA, HBsAg, HBsAb, HBeAg, and HBeAb, liver function tests, and hematology were measured on the 28th (baseline), 32nd, and 36th weeks of pregnancy, and on the 6th, 12th, 26th, 39th, and 52nd weeks, and 2nd, 3rd, and 4th years postpartum.

This research was approved by the Ethics Committee of the Beijing Ditan Hospital, Capital Medical University (jinglun dizi 2016–024 No. 01). Written and informed consent was obtained from all participants.

### 2.1. Statistical Analysis

Normally distributed continuous variables were expressed as mean ± standard deviation. Non-normally distributed quantitative data were presented as median and range. The non-parametric statistics, Chi-square test, and Fisher's exact test were used for comparison between the groups using the software IBM SPSS Statistics for mac version 26.0 (IBM Corp., Armonk, NY, USA). Clinical correlates were assessed by the Pearson Correlation test. Two-tailed and *P* value <0.05 was considered statistical significance.

## 3. Results

### 3.1. Baseline Characteristics

**Figure 1:**
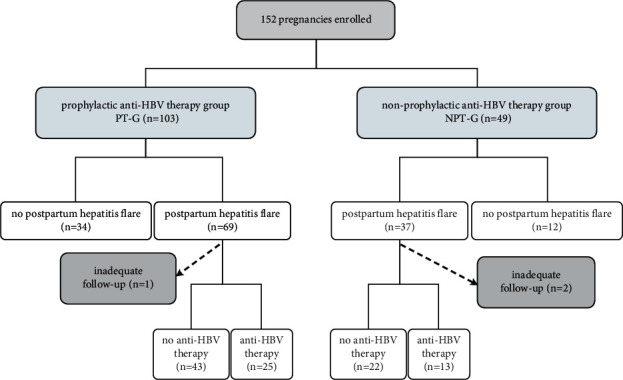
Summary of the patient selection process.

Figure 1 shows the pregnant women's selection process.  Table 1 presents the baseline demographic and clinical characteristics. All participants were HBeAg positive, with median age of 28.9 years and a median baseline HBV DNA log of 7.9 IU/mL. The age, parity, BMI, and levels of HBV DNA, HBeAg, HBcAb, HGB, PLT, ALT, AST, TBIL, and DBIL (*P* > 0.05) in the two groups (NPT-G vs. PT-G) were comparable. Only ALB levels were not comparable (*P*=0.003), revealing a statistically but not likely clinically significant difference. Of all anti-HBV pregnant women, 103 were treated with nucleoside analog (LdT, *n* = 96 and TDF, *n* = 7). The virological response was also different between PT-G and NPT-G, with a median reduction in viral load from the time of treatment initiation to delivery of 4.08 log·IU/mL in PT-G vs. 0.08 log·IU/mL in NPT-G (*P* ≤ 0.001).

### 3.2. Effects of Prophylactic Anti-HBV During the Third Trimester of Gestation on Postpartum Hepatitis Flare Recovery

**Figure 2:**
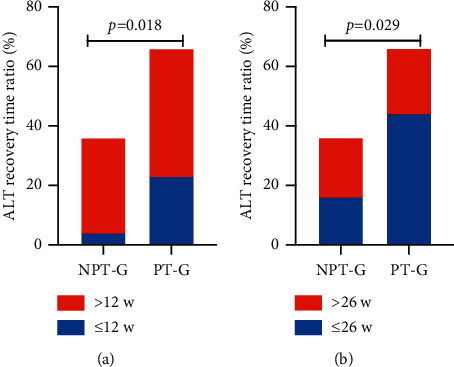
Comparison of ALT  recovery time postpartum in non-prophylactic anti-HBV therapy group (NPT-G) vs. prophylactic anti-HBV therapy group (PT-G). (a) ALT recovery time is bounded by 12 w (*x*^2^ = 5.579, *P*=0.018). (b) ALT recovery time is bounded by 26 w (*x*^2^ = 4.749, *P*=0.029).

**Figure 3:**
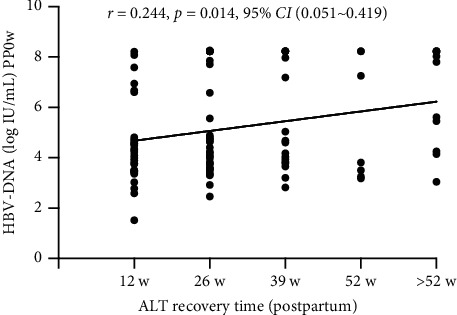
Association between ALT recovery time (postpartum) and HBV DNA (intrapartum, pp0 w) in women with postpartum hepatic flare. *r* = 0.244, *P*=0.014, 95% CI (0.051～0.419).

The postpartum flare rate in NPT-G and PT-G was 66.99% (69/103) and 75.51% (37/49), respectively; with no significant difference (*χ*^2^ = 2.31, *P*=0.138%). During the follow-up of all patients with postpartum hepatitis flare in the two groups, the ALT recovery rate was 11.1% (4/36) in NPT-G and 34.8% (23/66) in PT-G within postpartum 12 weeks (*P*=0.018; Table 1 and Figure 2(a)) and 44.4% (16/36) in NPT-G and 66.7% (44/66) in PT-G (*P*=0.029) within postpartum 26 weeks (Table 1 and Figure 2(b)). The ALT recovery rate was higher in PT-G than in NPT-G in 12 and 26 weeks. A weak correlation was found between HBV DNA quantification during delivery and ALT recovery postpartum (*r* = 0.244, *P*=0.014, 95% CI: 0.051–0.419; Figure 3).

### 3.3. Effects of Postpartum Anti-HBV Therapy on Hepatitis Flare Recovery and Hepatitis Re-Flare Within 4 Years Postpartum

**Figure 4:**
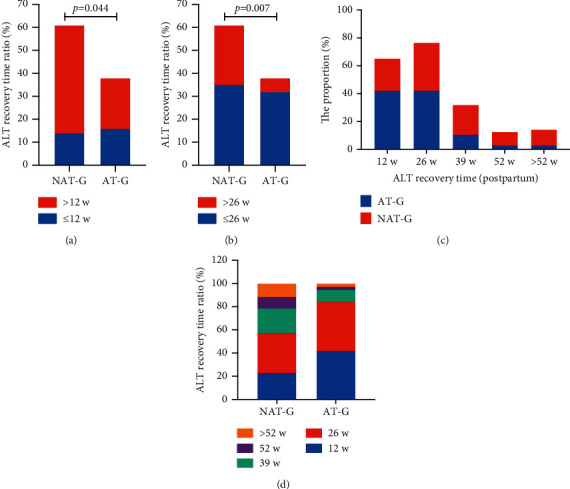
Comparison of ALT  recovery time postpartum in non-anti-HBV therapy group (NAT-G) vs. anti-HBV therapy group (AT-G). (a) ALT recovery time is bounded by 12 w (*x*^2^ = 4.067, *P*=0.044). (b) ALT recovery time is bounded by 26 w (*x*^2^ = 7.707, *P*=0.006). (c–d) Schematic diagram of the proportion of ALT  recovered within 1 year after delivery of the women with postpartum hepatitis flare (AT-G vs. NAT-G).

The incidence of postpartum hepatitis flare was 69% (106/152); 3 women had an inadequate follow-up. A total of 103 participants were divided into anti-HBV therapy group (AT-G, 38 women, including 4 women who did not stop prophylactic anti-HBV therapy during postpartum, 13 women who started anti-HBV after delivery, and 21 women with cessation of therapy after delivery and re-anti-HBV therapy during ALT flare) and non-anti-HBV therapy group (NAT-G, 65 women). The age, WBC count, and levels of HGB, PLT, HBV DNA, HBeAg, HBcAb, ALT, TBIL, and DBIL were not significantly different between the two groups at intrapartum. During the follow-up, the ALT recovery rate was 23% (14/65) in NAT-G and 42% (16/38) in AT-G (*χ*^2^ = 4.067, *P*=0.044) in 12 weeks (Table 2 and Figure 4(a)), 57.3% (35/65) in NAT-G and 84.2% (32/38) in AT-G (*χ*^2^ = 7.707, *P*=0.006) in 26 weeks (Table 2 and Figure 4(b)), and 88.5% (58/65) in NAT-G and 97.4% (37/38) in AT-G (*χ*^2^ = 1.226, *P*=0.268) in 52 weeks. The ALT recovery rate was higher in AT-G than in NAT-G in 12 and 26 weeks. However, no significant difference was observed in the recovery rate in 52 weeks. Table 2 and Figures 4(c) and 4(d) present the ratio of ALT recovery time in the two groups.

**Figure 5:**
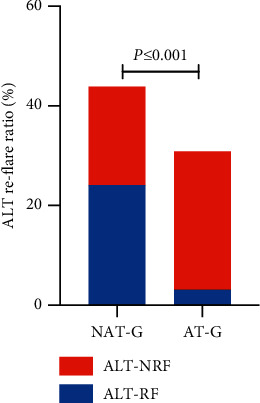
The proportion of ALT re-flare within 4 years postpartum of the women infected with HBV. The anti-HBV therapy group for postpartum hepatitis flare (AT-G) vs. non-anti-HBV therapy group for postpartum hepatitis flare (NAT-G) (*x*^2^ = 14.003, *P* ≤ 0.001). ALT-RF, ALT re-flare; ALT-NRF, ALT no re-flare.

The rates of hepatitis re-flare within 4 years postpartum was 54.5% (24/44) in NAT-G and 9.68% (3/28) in AT-G. The rate of hepatitis re-flare in NAT-G was significantly higher than that in AT-G (*χ*^2^ = 14.003, *P* ≤ 0.001; Table 2 and Figure 5).

### 3.4. Characteristics of Women within 4 Years Postpartum

**Figure 6:**
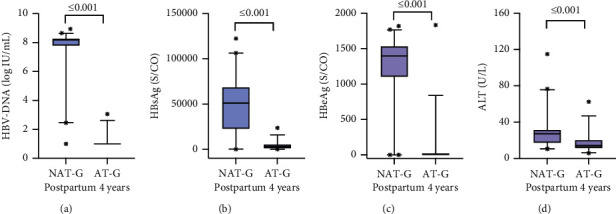
Comparison of HBV DNA, HBsAg, HBeAg, and ALT levels between two groups (NAT-G vs. AT-G). *P* < 0.05 was statistically significant, and the *P* value was shown above the horizontal line. (a) The HBV DNA level. (b) The HBsAg level. (c) The HBeAg level. (d) The ALT level.

**Figure 7:**
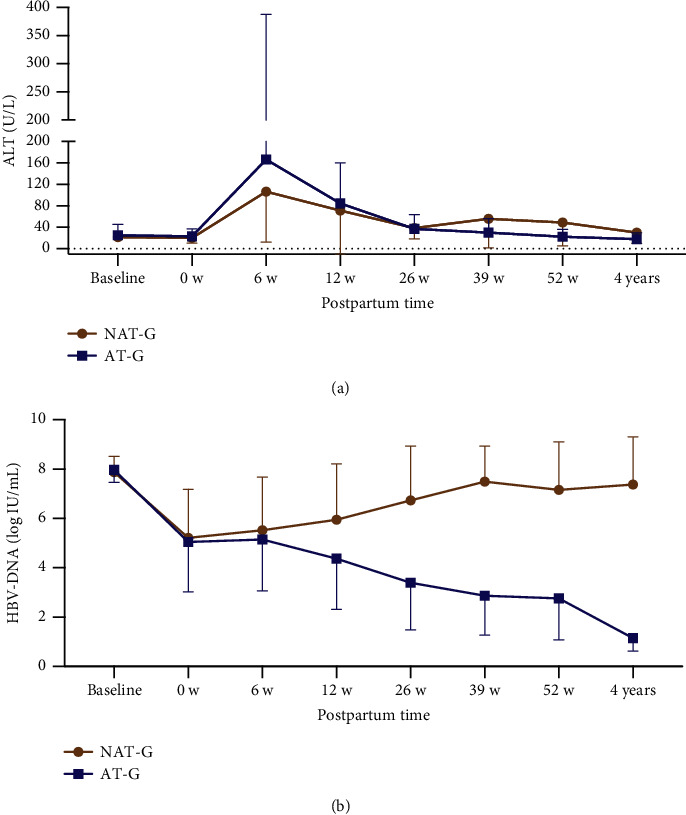
ALT (a) and HBV DNA (b) trends from baseline to delivery, and 4 years postpartum.

Both groups (NAT-G and AT-G) were followed up at least once during the postpartum second, third, and fourth years, and their virology and ALT were compared during 4 years postpartum. HBeAg loss rate and the proportion of HBV DNA below the detection limit (<100 IU/mL) in AT-G were significantly higher than those in NAT-G (*P* < 0.05). The HBeAg, HBsAg, HBV DNA, and ALT levels in AT-G were significantly lower than those in NAT-G (*P* < 0.001) during 4 years postpartum (Table 3 and Figure 6). ALT and HBV DNA trends from baseline to intrapartum and 1–4 years postpartum are shown in Figure 7.

## 4. Discussion

No clear conclusion exists in the international guidelines on whether HBV-infected women need postpartum treatment for hepatitis flare. Also, studies exploring the clinical benefit of continuous anti-HBV therapy for women with postpartum hepatitis flare and the long-term prognosis for women without anti-HBV therapy are limited. Postpartum hepatitis flare is more common among women with HBV infection, with an incidence rate between 25 and 44.7% [4–7]. Most studies found that nucleoside analog therapy in late pregnancy, followed by treatment withdrawal early postpartum, was associated with ALT flare; however, they reported variable changes in HBV DNA levels or rates of HBeAg seroconversion during immediate postpartum up to 1 year of follow-up [8, 11, 14–16]. The definition of a hepatitis flare varies according to the literature [7, 17, 18], but a significant increase in serum ALT levels from the baseline level or higher than ULN is generally defined as ALT flare [10]. In this observational study, the rate of postpartum ALT flare was 69%. Two main reasons accounted for the variable rates of ALT flares. First, it was related to HBV DNA viral load and the positivity of HBsAg. The HBsAg-positive population had a higher rate of postpartum hepatitis flare [5, 9]. All pregnant women in this observational study were HBsAg positive. Second, it was related to the standard of postpartum hepatitis flare. When the cutoff value of ALT was low, the proportion of postpartum ALT flare was correspondingly higher. Overall, the percentage of postpartum ALT flare was not low. However, few studies examined its long-term outcomes.

Altered immune status during pregnancy in patients with chronic HBV infection, result in a sudden decrease in cortisol levels in postpartum, which is similar to the withdrawal of steroid therapy, and may lead to HBV reactivation and inflammatory response [5, 19–21]. Furthermore, reducing the HBsAg/HBeAg antigen burden through anti-HBV therapy may lead to the recovery of T cell exhaustion, enhance HBV-specific immune-mediated killing of HBV-infected cells, and improve immune control [22]. The most recent meta-analysis on this topic showed that lamivudine, telbivudine, and TDF could be used safely without increasing the risk of postpartum hepatitis flares after the treatment withdrawal [23]. In addition, among women treated with tenofovir, available data showed that HBV DNA load decline significantly in women with a postpartum ALT flare than in those with no ALT flare from baseline [24]. Therefore, postpartum hepatitis flare may be an opportunity for anti-HBV treatment.

Postpartum 1 month (4 w) to 3 months (12 w) is the time of high incidence of hepatitis flare, most of the maternal ALT flares return to normal in 3 months (12 w) to 6 months (26 w). In this study, the rate of postpartum hepatitis flare recovery within 12 and 26 weeks was significantly higher in the anti-HBV therapy group than in the non-anti-HBV therapy group. In addition, 7 (11.4%) patients in the non-anti-HBV therapy group with postpartum hepatitis flares and only 1 (2.6%) in the anti-HBV therapy group did not recover within 1 year postpartum. Another finding of this study that supported postpartum anti-HBV therapy was that the rate of postpartum hepatitis re-flare for the long term in the anti-HBV group was significantly lower (9.6%) than that (54.5%) in the non-anti-HBV therapy group. These results suggested that postpartum anti-HBV therapy was beneficial in controlling recurrent hepatitis flares.

Women who had hepatitis flare within 26 weeks postpartum but did not receive anti-HBV treatment were more likely to have hepatitis re-flare after 26 weeks postpartum compared with women who did not have hepatitis flare within 26 weeks postpartum. The results indicated that anti-HBV treatment could help improve the liver condition by maintaining the normal ALT level, and anti-HBV therapy could prevent the incidence of hepatitis re-flare. Repeated hepatitis flare reflected the repeated activity of liver inflammation and was closely related to chronic liver fibrosis. In terms of long-term prognosis, reducing hepatitis re-flare could prevent the formation of liver fibrosis.

HBsAg clearance and HBeAg loss occurred at an earlier time in patients who started anti-HBV treatment in pregnancy compared with untreated patients (HBsAg loss was noted at a median of 2 vs. 2.5 years, and HBeAg loss at a median of 1 vs. 3 years) [24]. In this study, during 4 years of postpartum follow-up, the ALT level in the postpartum anti-HBV therapy group was lower than that in the non-anti-HBV therapy group. The absence of liver elasticity measurements in 4 years did not reflect the difference in liver fibrosis between the postpartum anti-HBV and non-anti-HBV groups. This was the limitation of this study. Other limitations were that it was a retrospective study, the sample size was limited, and the postpartum follow-up was difficult, especially during a follow-up period of 1 year later.

## 5. Conclusion

The results of this study suggested that postpartum hepatitis flare was still common, and anti-HBV therapy could shorten the time needed for postpartum hepatitis flare recovery and reduce the incidence of hepatitis re-flare. Women with postpartum hepatitis flare were at a higher risk of re-flare than those without postpartum hepatitis flare. To some extent, anti-HBV therapy for postpartum hepatitis flare is beneficial. However, the long-term prognosis needs to be evaluated over longer follow-up periods. After the withdrawal of prophylactic anti-HBV therapy, clinicians should carefully monitor for the occurrence of hepatitis flare. Hepatitis flare recovered spontaneously in 88.5% of women within 1 year of postpartum, but hepatitis flare in a small percentage of women lasted longer than a year. A closing follow-up is strongly recommended to initiate anti-HBV therapy if necessary.

## Figures and Tables

**Table 1:** Baeseline characteristics of PT-G and NPT-G.

Baeseline characteristics	Group 1: NPT-G (*n* = 49) M (P25, P75)	Group 2: PT-G (*n* = 103) M (P25, P75)	t/Z/x^2^	*P*
Age (years)	28 (26, 30.5)	29 (27, 32)	1.545	0.122
BMI (kg/m^2^)	23.91 (21.94, 26.02)	24.84 (23.05, 26.95)	1.592	0.114
WBC (×10^9^/L)	8.60 (7.45, 9.70)	8.91 (7.89, 9.90)	1.330	0.184
HGB (g/L)	118 (112, 125)	121 (113, 126)	1.325	0.185
PLT (×10^9^/L)	194.00 (170.00, 228.00)	197.50 (171.75, 226.75)	0.427	0.669
ALT (U/L)	18.50 (14.50, 23.00)	19.50 (14.20, 25.70)	0.320	0.749
AST (U/L)	18 (17.10, 22.00)	19.10 (15.90, 7.90)	0.144	0.886
TBIL (umol/L)	6.50 (5.40, 7.90)	6.00 (5.20, 7.90)	−0.904	0.336
DBIL (umol/L)	1.50 (1.10, 1.90)	1.40 (1.10, 1.80)	−0.729	0.466
ALB (g/L)	38.20 (1325.80, 1706.70)	37.00 (35.30, 38.70)	−2.974	0.003
HBeAg (S/CO)	1509.60 (1325.80, 1706.70)	1543.16 (1376.86, 1696.36)	0.394	0.693
HBcAb (S/CO)	9.89 (8.47, 10.99)	9.71 (8.78, 10.49)	−0.224	0.823

HBV DNA (log IU/mL)				
Baseline	8.23 (7.68, 8.23)	8.23 (7.94, 8.23)	−0.650	0.516
Intrapartum	8.20 (7.38, 8.23)	3.81 (3.36, 4.54)		
HBV DNA median fall	0.08 (0.00, 0.48)	4.08 (3.52, 4.58)	8.687	≤0.001

Parity			1.541	0.214
Primiparity	*n* = 19 (38.78%)	*n* = 51 (49.51%)		
multiparity	*n* = 30 (61.22%)	*n* = 52 (50.49%)		

Type of anti-HBV therapy				
Telbivudine		*n* = 96 (93.20%)		
Tenofovir		*n* = 7 (6.80%)		
Postpartum hepatitis flare	37 (75%)	71 (69%)		
No postpartum hepatitis flare	12 (24%)	32 (31%)		

ALT recovery time (postpartum hepatitis flare women)				
≤12 w	4 (11.1%)	23 (34.8%)	x^2^ = 5.579	0.018
>12 w	32 (88.8%)	43 (65.2%)		
≤26 w	16 (44.4%)	44 (66.7%)	*x* ^2^ = 4.749	0.029
>26 w	20 (55.6%)	22 (33.3%)		

ALT, alanine aminotransferase; AST, aspartate aminotransferase; TBil, total bilirubin; DBIL, direct bilirubin; HBeAg, hepatitis B e antigen; HBsAg, hepatitis B surface antigen; HBV, hepatitis B virus; HBV DNA, hepatitis B deoxyribonucleic acid; SD, standard deviation; ULN, upper limit of normal; WBC, white blood cell; PLT, platelet; HGB, hemoglobin; ALB, albumin.

**Table 2:** Comparison between AT-G and NAT-G.

intrapartum characteristics	NAT-G (*n* = 65) *n* (%), M (P25, P75)	AT-G (*n* = 38) *n* (%), M (P25, P75)	Z/x^2^	*P*
Age (years)	29 (26, 31.5)	30 (27, 31.5)	Z = 0.576	0.564
WBC (×10^9^/L)	8.49 (6.84, 9.83)	7.86 (6.92, 9.88)	Z = 0.277	0.782
HGB (g/L)	126 (118, 133)	124 (112, 132.25)	Z = 0.523	0.601
PLT (×10^9^/L)	188 (167.5, 223.5)	178 (155.25, 208.75)	Z = −1.517	0.129
ALT (U/L)	17.7 (13.03, 26.45)	20.75 (14.55, 28.48)	Z = 1.267	0.205
ALT (U/L) PP 6 w	62.7 (45.23, 126.05)	89.5 (59.88, 174.93)	Z = 1.864	0.062
ALT (U/L) PP 12 w	51.1 (35.1, 78.13)	61.3 (28.7, 117.4)	Z = 1.056	0.291
AST (U/L)	20 (16.8, 26.15)	21.15 (17.55, 28.280	Z = 1.28	0.200
TBIL (umol/L)	7.2 (5.63, 8.4)	6.85 (5.23, 8.73)	Z = 0.699	0.484
DBIL (umol/L)	1.5 (1.2, 2.1)	1.65 (1.28, 2.03)	Z = 0.926	0.355
ALB (g/L)	37 (34.7, 38.28)	34.9 (32.72, 37.13)	Z = 3.268	0.001
HBeAg (S/CO)	1510.53 (1304.9, 1633.23)	1517.88 (1184.97, 1633.35)	Z = 0.199	0.842
HBcAb (S/CO)	9.86 (8.58, 10.52)	10.12 (9.17, 10.70)	Z = 0.806	0.421
HBV DNA (log IU/mL)	4.46 (3.81, 7.60)	4.27 (3.40, 7.36)	Z = −0.914	0.361

ALT recovery time				
≤12 week	14 (23.0%)	16 (42.1%)	x^2^ = 4.067	0.044
>12 week	47 (77.0%)	22 (57.9%)		
≤26 week	35 (57.3%)	32 (84.2%)	x^2^ = 7.707	0.006
>26 week	26 (42.6%)	6 (15.8%)		

ALT re-flare within 4 years				
Re-flare (ALT-RF)	24 (45.4%)	3 (9.68%)	x^2^ = 14.003	≤0.001
No re-flare (ALT-NRF)	20 (54.6%)	28 (90.32%)		

PP 6 w, postpartum 6 weeks; PP 12 w, postpartum 12 weeks; ALT, alanine aminotransferase; AST, aspartate aminotransferase; TBil, total bilirubin; DBIL, direct bilirubin; HBeAg, hepatitis B e antigen; HBsAg, hepatitis B surface antigen; HBV, hepatitis B virus; HBV DNA, hepatitis B deoxyribonucleic acid; SD, standard deviation; WBC, white blood cell; PLT, platelet; HGB, hemoglobin.

**Table 3:** Prognosis of the women postpartum 4 years.

characteristics	NAT-G (*n* = 46) *n*/%, M (P25, P75)	AT-G (*n* = 38) *n*/%, M (P25, P75)	Z/x^2^	*P*
ALT (U/L)	16.6 (11.65, 27.8)	27.4 (15.15, 36.65)	4.065	≤0.001
HBsAg (S/CO)	53786 (29982.49, 69098.77)	2712.85 (847.54, 4675.69)	5.527	≤0.001
HBeAg (S/CO)	1398 (1100, 33)	3.6 5 (0.39, 23.07)	6.123	≤0.001
HBeAg loss proportion	*n* = 2 (4.34%)	*n* = 7 (18.91%)	4.599	0.040
HBV DNA (log IU/mL)	8.23 (7.78, 8.23)	<2 (<Detectionlimit)	7.941	≤0.001
HBV DNA<Detectionlimit	*n* = 1 (2.17%)	*n* = 35 (92.1%)	68.723	≤0.001

ALT, alanine aminotransferase; HBeAg, hepatitis B e antigen; HBsAg, hepatitis B surface antigen; HBV DNA, hepatitis B deoxyribonucleic acid.

## Data Availability

The datasets used and/or analyzed during the current study are available from the corresponding author upon reasonable request.

## Supplementary Materials

Supplementary Materials Supplementary Table 1 is a description of the abbreviations in the manuscript. Supplementary Table 2 is the follow-up table of the study subjects.

## References

[1] Seto W. K., Lo Y. R., Pawlotsky J. M., Yuen M. F. (2018). Chronic hepatitis B virus infection. *The Lancet*.

[2] Hyun M. H., Lee Y. S., Kim J. H. (2017). Systematic review with meta-analysis: the efficacy and safety of tenofovir to prevent mother-to-child transmission of hepatitis B virus. *Alimentary Pharmacology & Therapeutics*.

[3] Chen H. L., Zha M. L., Cai J. Y., Qin G. (2018). Maternal viral load and hepatitis B virus mother-to-child transmission risk: a systematic review and meta-analysis. *Hepatology Research*.

[4] Han G. R., Cao M. K., Zhao W. (2011). A prospective and open-label study for the efficacy and safety of telbivudine in pregnancy for the prevention of perinatal transmission of hepatitis B virus infection. *Journal of Hepatology*.

[5] Giles M., Visvanathan K., Lewin S. (2015). Clinical and virological predictors of hepatic flares in pregnant women with chronic hepatitis B. *Gut*.

[6] Pan C. Q., Duan Z., Dai E. (2016). Tenofovir to prevent hepatitis B transmission in mothers with high viral load. *New England Journal of Medicine*.

[7] Liu J., Wang J., Jin D. (2017). Hepatic flare after telbivudine withdrawal and efficacy of postpartum antiviral therapy for pregnancies with chronic hepatitis B virus. *Journal of Gastroenterology and Hepatology*.

[8] Nguyen V., Tan P. K., Greenup A. J. (2014). Anti-viral therapy for prevention of perinatal HBV transmission: extending therapy beyond birth does not protect against post-partum flare. *Alimentary Pharmacology & Therapeutics*.

[9] Kushner T., Shaw P. A., Kalra A. (2018). Incidence, determinants and outcomes of pregnancy-associated hepatitis B flares: a regional hospital-based cohort study. *Liver International*.

[10] Bzowej N. H., Tran T. T., Li R. (2019). Total alanine aminotransferase (ALT) flares in pregnant north American women with chronic hepatitis B infection: results from a prospective observational study. *American Journal of Gastroenterology*.

[11] Chang C. Y., Aziz N., Poongkunran M. (2016). Serum alanine aminotransferase and hepatitis B DNA flares in pregnant and postpartum women with chronic hepatitis B. *American Journal of Gastroenterology*.

[12] Nguyen G., Garcia R. T., Nguyen N., Trinh H., Keeffe E. B., Nguyen M. H. (2009). Clinical course of hepatitis B virus infection during pregnancy. *Alimentary Pharmacology & Therapeutics*.

[13] Singhal A., Kanagala R., Jalil S., Wright H. I., Kohli V. (2011). Chronic HBV with pregnancy: reactivation flare causing fulminant hepatic failure. *Annals of Hepatology*.

[14] Wang M., Bian Q., Zhu Y. (2019). Real-world study of tenofovir disoproxil fumarate to prevent hepatitis B transmission in mothers with high viral load. *Alimentary Pharmacology & Therapeutics*.

[15] Chang K. C., Chang M. H., Lee C. N. (2019). Decreased neonatal hepatitis B virus (HBV) viremia by maternal tenofovir treatment predicts reduced chronic HBV infection in children born to highly viremic mothers. *Alimentary Pharmacology & Therapeutics*.

[16] Virine B., Osiowy C., Gao S. (2015). Hepatitis B virus (HBV) variants in untreated and tenofovir treated chronic hepatitis B (CHB) patients during pregnancy and post-partum follow-up. *PLoS One*.

[17] Chang C. Y., Aziz N., Poongkunran M. (2018). Serum aminotransferase flares in pregnant and postpartum women with current or prior treatment for chronic hepatitis B. *Journal of Clinical Gastroenterology*.

[18] Lee Y. S., Bang S. M., Lee Y. S. (2021). Benefits and risks of antiviral treatment during pregnancy in patients with chronic hepatitis B. *Journal of Clinical Medicine*.

[19] ter Borg M. J., Leemans W. F., de Man R. A., Janssen H. L. A. (2008). Exacerbation of chronic hepatitis B infection after delivery. *Journal of Viral Hepatitis*.

[20] Huang M., Gao Y., Yin X. (2021). Characterization of T cell immunity in chronic hepatitis B virus-infected mothers with postpartum alanine transaminase flare. *BMC Infectious Diseases*.

[21] Joshi S. S., Coffin C. S. (2020). Hepatitis B and pregnancy: virologic and immunologic characteristics. *Hepatol Commun*.

[22] Rinker F., Zimmer C. L., Höner Zu Siederdissen C. (2018). Hepatitis B virus-specific T cell responses after stopping nucleos (t)ide analogue therapy in HBeAg-negative chronic hepatitis B. *Journal of Hepatology*.

[23] Funk A. L., Lu Y., Yoshida K. (2021). Efficacy and safety of antiviral prophylaxis during pregnancy to prevent mother-to-child transmission of hepatitis B virus: a systematic review and meta-analysis. *The Lancet Infectious Diseases*.

[24] Samadi Kochaksaraei G., Castillo E., Sadler M. D. (2020). Real-world clinical and virological outcomes in a retrospective multiethnic cohort study of 341 untreated and tenofovir disoproxil fumarate-treated chronic hepatitis B pregnant patients in North America. *Alimentary Pharmacology and Therapeutics*.

