# Supplementary material for: Antiviral Therapy for a Postpartum Flare in Women with Chronic HBV Infection Shortens the ALT Recovery Time and Reduces Hepatitis Re-Flare Rates within 4 years
Source: Can J Gastroenterol Hepatol. 2022 Jun 20;2022:4753267. doi: 10.1155/2022/4753267 (PMC9236834; doi:10.1155/2022/4753267)
Supplement: Supplementary Materials — Supplementary Table 1 is a description of the abbreviations in the manuscript. Supplementary Table 2 is the follow-up table of the study subjects. [file 4753267.f1.zip › 4753267.f1/Supplement table 1.pdf]

Supplement table 1

abbreviations	
PT-G	prophylactic anti-HBV therapy group
NPT-G	non-prophylactic anti-HBV therapy group
AT-G	anti-HBV therapy group
NAT-G	non-anti-HBV therapy group
ALT-NRF	ALT no re-flare
ALT-RF	ALT re-flare
