# Supplementary figures and images for: Antiviral Therapy for a Postpartum Flare in Women with Chronic HBV Infection Shortens the ALT Recovery Time and Reduces Hepatitis Re-Flare Rates within 4 years
Source: Can J Gastroenterol Hepatol. 2022 Jun 20;2022:4753267. doi: 10.1155/2022/4753267 (PMC9236834; doi:10.1155/2022/4753267)

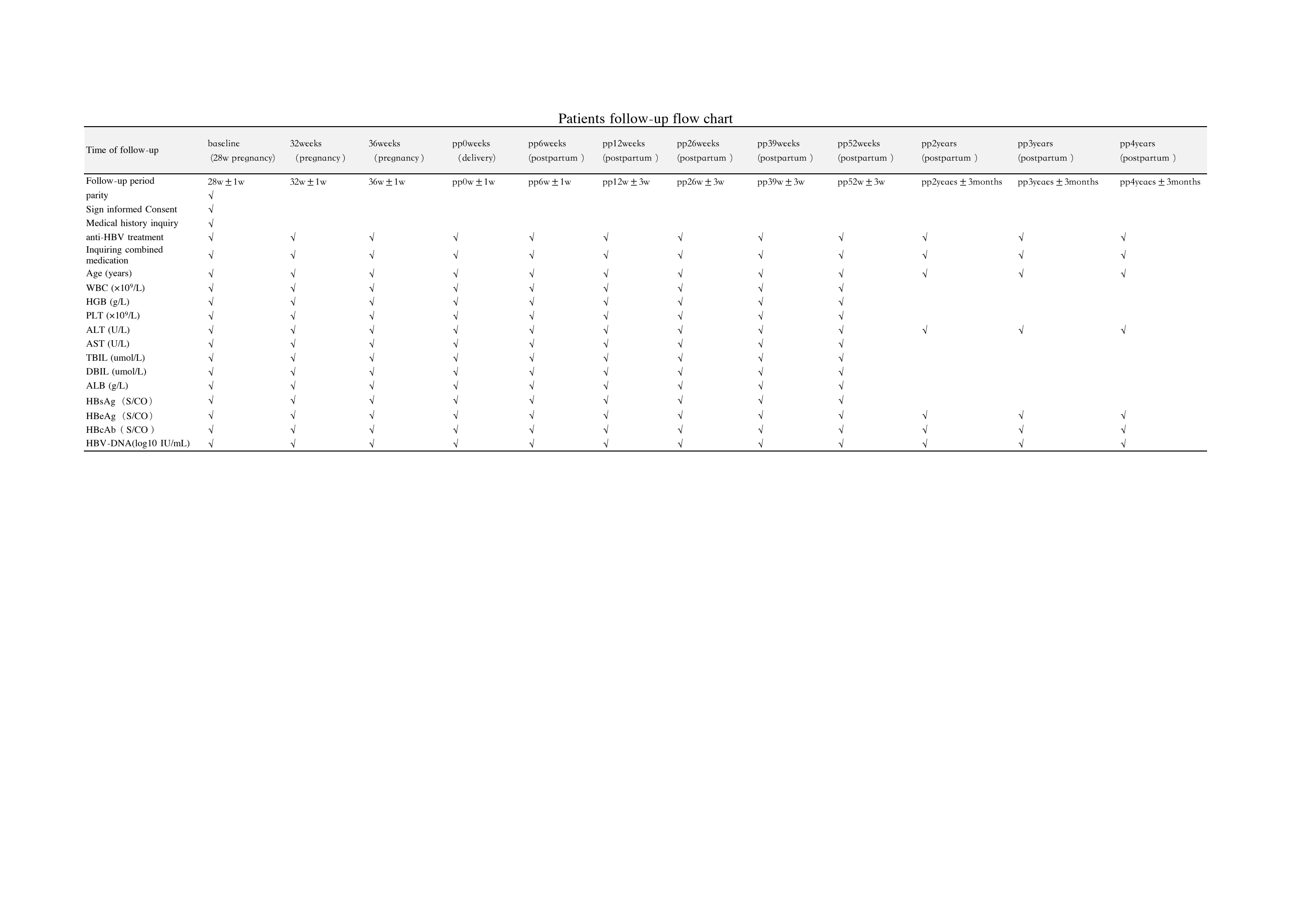

Supplement: Supplementary Materials — Supplementary Table 1 is a description of the abbreviations in the manuscript. Supplementary Table 2 is the follow-up table of the study subjects. [file 4753267.f1.zip › 4753267.f1/supplement table2(follow up flow chart).png]
